# Supplementary material for: Gene Loss DB: a curated database for gene loss in mammals—the cetacean collection
Source: Database (Oxford). 2025 Sep 24;2025:baaf065. doi: 10.1093/database/baaf065 (PMC12462620; doi:10.1093/database/baaf065)
Supplement: baaf065_Supplemental_Files [file baaf065_supplemental_files.zip › Supplementary Table 1_Revised.pdf]

Supplementary Table 1: Curated publications

Title	Nr. of GL	DOI
Minke whale genome and aquatic adaptation in cetaceans.	6	10.1038/ng.2835
Functional or Vestigial? The Genomics of the Pineal Gland in Xenarthra.	98	10.1007/s00239-021-10025-1
Parallel Independent Losses of G-Type Lysozyme Genes in Hairless Aquatic Mammals.	98	10.1093/gbe/evab201
Recurrent loss of HMGCS2 shows that ketogenesis is not essential for the evolution of large mammalian brains.	23	10.7554/eLife.38906
Gene duplications and gene loss in the epidermal differentiation complex during the evolutionary land-to-water transition of cetaceans.	79	10.1038/s41598-021-91863-3
Analysis of the FGF gene family provides insights into aquatic adaptation in cetaceans.	9	10.1038/srep40233
Complete Inactivation of Sebum-Producing Genes Parallels the Loss of Sebaceous Glands in Cetacea.	67	10.1093/molbev/msz068
Genomewide analysis of sperm whale E2 ubiquitin conjugating enzyme genes.	4	10.1007/s12041-021-01333-y
Molecular decay of the tooth gene Enamelin (ENAM) mirrors the loss of enamel in the fossil record of placental mammals.	20	10.1371/journal.pgen.1000634
Comparative genomics of sirenians reveals evolution of filaggrin and caspase-14 upon adaptation of the epidermis to aquatic life.	10	10.1038/s41598-024-60099-2
Epidermal cornification is preceded by the expression of a keratinocyte-specific set of pyroptosis-related genes.	62	10.1038/s41598-017-17782-4
Genetic basis of brain size evolution in cetaceans: insights from adaptive evolution of seven primary microcephaly (MCPH) genes.	7	10.1186/s12862-017-1051-7
Genetic evidence for the ancestral loss of short-wavelength-sensitive cone pigments in mysticete and odontocete cetaceans.	16	10.1098/rspb.2002.2278
Ancient convergent losses of yield potential risks for modern marine mammals.	10	10.1126/science.aap7714
A genomics approach reveals insights into the importance of gene losses for mammalian adaptations.	111	10.1038/s41467-018-03667-1
Positive Selection and Inactivation in the Vision and Hearing Genes of Cetaceans.	42	10.1093/molbev/msaa070
Progressive erosion of the Relaxin1 gene in bovids.	43	10.1016/j.ygcen.2017.07.011
Comparative genomics provides insights into the aquatic adaptations of mammals.	25	10.1073/pnas.2106080118
Inactivation of thermogenic UCP1 as a historical contingency in multiple placental mammal clades.	31	10.1126/sciadv.1602878
Convergent Loss of the Necroptosis Pathway in Disparate Mammalian Lineages Shapes Viruses Countermeasures.	65	10.3389/fimmu.2021.747737
Morphological and molecular evidence for a stepwise evolutionary transition from teeth to baleen in mysticete whales.	11	10.1080/10635150701884632

Unusual loss of chymosin in mammalian lineages parallels neo-natal immune transfer strategies.	30	10.1016/j.ympev.2017.08.014
Patterns and tempo of PCSK9 pseudogenizations suggest an ancient divergence in mammalian cholesterol homeostasis mechanisms.	165	10.1007/s10709-021-00113-x
The Novel Evolution of the Sperm Whale Genome.	19	10.1093/gbe/evx187
Convergent inactivation of the skin-specific C-C motif chemokine ligand 27 in mammalian evolution.	18	10.1007/s00251-019-01114-z
Evolution of bitter taste receptors in humans and apes.	47	10.1093/molbev/msi027
Evolution of the MC5R gene in placental mammals with evidence for its inactivation in multiple lineages that lack sebaceous glands.	17	10.1016/j.ympev.2017.12.010
The dopamine receptor D gene shows signs of independent erosion in toothed and baleen whales.	14	10.7717/peerj.7758
The Singularity of Cetacea Behavior Parallels the Complete Inactivation of Melatonin Gene Modules.	49	10.3390/genes10020121
Genes lost during the transition from land to water in cetaceans highlight genomic changes associated with aquatic adaptations.	266	10.1126/sciadv.aaw6671
Increased rate of hair keratin gene loss in the cetacean lineage.	28	10.1186/1471-2164-15-869
Convergent Losses of TLR5 Suggest Altered Extracellular Flagellin Detection in Four Mammalian Lineages.	23	10.1093/molbev/msaa058
Regressed but Not Gone: Patterns of Vision Gene Loss and Retention in Subterranean Mammals.	360	10.1093/icb/icy004
Inactivation of the olfactory marker protein (OMP) gene in river dolphins and other odontocete cetaceans.	12	10.1016/j.ympev.2017.01.020
Cetacea are natural knockouts for IL20.	11	10.1007/s00251-018-1071-5
A drastic shift in the energetic landscape of toothed whale sperm cells.	150	10.1016/j.cub.2021.05.062
Molecular evolutionary analyses of tooth genes support sequential loss of enamel and teeth in baleen whales (Mysticeti).	130	10.1016/j.ympev.2022.107463
Phylogenetic profiling and gene expression studies implicate a primary role of PSORS1C2 in terminal differentiation of keratinocytes.	11	10.1111/exd.13272
Insights into the evolution of longevity from the bowhead whale genome.	16	10.1016/j.celrep.2014.12.008
Whale Hageman factor (factor XII): prevented production due to pseudogene conversion.	1	10.1016/s0049-3848(97)00307-1
GBA3: a polymorphic pseudogene in humans that experienced repeated gene loss during mammalian evolution.	35	10.1038/s41598-020-68106-y
Aquatic adaptation and the evolution of smell and taste in whales.	61	10.1186/s40851-014-0002-z
Pseudogenization of the tooth gene enamelysin (MMP20) in the common ancestor of extant baleen whales.	9	10.1098/rspb.2010.1280
Major taste loss in carnivorous mammals.	12	10.1073/pnas.1118360109
Birth-and-death evolution of ribonuclease 9 genes in Cetartiodactyla.	59	10.1007/s11427-022-2195-x
Rod monochromacy and the coevolution of cetacean retinal opsins.	34	10.1371/journal.pgen.1003432
The loss of taste genes in cetaceans.	75	10.1186/s12862-014-0218-8
Mx1 and Mx2 key antiviral proteins are surprisingly lost in toothed whales.	8	10.1073/pnas.1501844112
Losses of human disease-associated genes in placental mammals.	100	10.1093/nargab/lqz012
Genomic and anatomical comparisons of skin support independent adaptation to life in water by cetaceans and hippos.	230	10.1016/j.cub.2021.02.057

Transition to an Aquatic Habitat Permitted the Repeated Loss of the Pleiotropic KLK8 Gene in Mammals.	9	10.1093/gbe/evx239
Loss or major reduction of umami taste sensation in pinnipeds.	7	10.1007/s00114-012-0939-8
Decay of Skin-Specific Gene Modules in Pangolins.	50	10.1007/s00239-023-10118-z
Convergent Cortistatin losses parallel modifications in circadian rhythmicity and energy homeostasis in Cetacea and other mammalian lineages.	63	10.1016/j.ygeno.2020.11.002
Rubbing Salt in the Wound: Molecular Evolutionary Analysis of Pain-Related Genes Reveals the Pain Adaptation of Cetaceans in Seawater.	9	10.3390/ani12243571
Comparative genomics analyses of alpha-keratins reveal insights into evolutionary adaptation of marine mammals.	210	10.1186/s12983-017-0225-x
