# Supplementary material for: Gene Loss DB: a curated database for gene loss in mammals—the cetacean collection
Source: Database (Oxford). 2025 Sep 24;2025:baaf065. doi: 10.1093/database/baaf065 (PMC12462620; doi:10.1093/database/baaf065)
Supplement: baaf065_Supplemental_Files [file baaf065_supplemental_files.zip › Supplementary table 2_revised.pdf]

Supplementary Table 2: Number of gloss annotations in Cetacea by species

Odontoceti	GLoss	Mysticeti	GLoss
Tursiops truncatus	268	*Balaenoptera acutorostrata*	269
Physeter catodon	242	*Balaena mysticetus*	82
Orcinus orca	215	*Balaenoptera bonaerensis*	56
Lipotes vexillifer	98	*Eschrichtius robustus*	39
Neophocaena asiaeorientalis	63	*Balaenoptera musculus*	36
Delphinapterus leucas	42	*Balaenoptera physalus*	24
Phocoena sinus	38	*Megaptera novaeangliae*	20
Sousa chinensis	30	*Caperea marginata*	16
Kogia sima	24	*Balaenoptera borealis*	15
Lagenorhynchus obliquidens	24	*Eubalaena japonica*	15
Kogia breviceps	22	*Eubalaena australis*	13
Monodon monoceros	23	*Eubalaena glacialis*	13
Globicephala melas	21	*Balaenoptera edeni*	12
Phocoena phocoena	17	*Balaenoptera brydei*	1
Neophocaena phocaenoides	12	*Balaenoptera omurai*	1
Pontoporia blainvillei	14		
Inia geoffrensis	14		
Tursiops aduncus	14		
Mesoplodon bidens	13		
Ziphius cavirostris	12		
Delphinus capensis	9		
Peponocephala electra	8		
Phocoenoides dalli	3		
Platanista gangetica	3		
Mesoplodon europaeus	3		
Platanista minor	5		
Mesoplodon densirostris	3		
Mesoplodon mirus	2		
Berardius bairdii	2		
Mesoplodon ginkgodens	2		
Mesoplodon carlhubbsi	2		
Mesoplodon stejnegeri	2		
Grampus griseus	2		
Tasmacetus shepherdi	1		
Pseudorca crassidens	1		
Mesoplodon perrini	1		
Globicephala macrorhynchus	1		
Lagenorhynchus australis	1		
Mesoplodon bowdoini	1		
Hyperoodon ampullatus	1		
Delphinus delphis	1		
